# Zeaxanthin Induces Apoptosis in Human Uveal Melanoma Cells through Bcl-2 Family Proteins and Intrinsic Apoptosis Pathway

**DOI:** 10.1155/2013/205082

**Published:** 2013-10-10

**Authors:** Ming-Chao Bi, Richard Rosen, Ren-Yuan Zha, Steven A. McCormick, E. Song, Dan-Ning Hu

**Affiliations:** ^1^Department of Ophthalmology, The First Hospital of Jilin University, 71 Xinmin Street, Changchun 130021, China; ^2^Department of Ophthalmology, The New York Eye and Ear Infirmary, New York Medical College, 310 E. 14th Street, New York, NY 10003, USA; ^3^Department of Oncological Sciences, Tisch Cancer Institute, Icahn School of Medicine at Mount Sinai, New York, NY 10029, USA; ^4^Tissue Culture Center, The New York Eye and Ear Infirmary, New York Medical College, New York, NY 10003, USA; ^5^Department of Pathology, The New York Eye and Ear Infirmary, New York Medical College, 310 E. 14th Street, New York, NY 10003, USA

## Abstract

The cytotoxic effects of zeaxanthin on two human uveal melanoma cell lines (SP6.5 and C918) and related signaling pathways were studied and compared to effects on normal ocular cells (uveal melanocytes, retinal pigment epithelial cells, and scleral fibroblasts). MTT assay revealed that zeaxanthin reduced the cell viability of melanoma cells in a dose-dependent manner (10, 30, and 100 **μ**M), with IC_50_ at 40.8 and 28.7 **μ**M in SP6.5 and C918 cell lines, respectively. Zeaxanthin did not affect the viability of normal ocular cells even at the highest levels tested (300 **μ**M), suggesting that zeaxanthin has a selectively cytotoxic effect on melanoma cells. Zeaxanthin induced apoptosis in melanoma cells as indicated by annexin V and ethidium III flow cytometry. Western blot analysis demonstrated that zeaxanthin decreased the expression of antiapoptotic proteins (Bcl-2 and Bcl-xL) and increased the expression of proapoptotic proteins (Bak and Bax) in zeaxanthin-treated melanoma cells. Zeaxanthin increased mitochondrial permeability as determined by JC-1 fluorescein study. Zeaxanthin also increased the level of cytosol cytochrome c and caspase-9 and -3 activities, but not caspase-8, as measured by ELISA assay or colorimetric assay. All of these findings indicate that the intrinsic (mitochondrial) pathway is involved in zeaxanthin-induced apoptosis in uveal melanoma cells.

## 1. Introduction

Uveal melanoma is the most common primary intraocular tumor in the adult population, with an incidence of 6-7 cases per million per year in the US [1]. Uveal melanoma has a poor prognosis, with half of uveal melanoma patients dying from their disease within 25 years [2]. The high mortality rate is related to the development of metastasis, which has a strong preference for the liver. Most uveal melanoma patients with liver metastasis die within 6 months [2–4]. Because of the poor prognosis of metastatic uveal melanoma, new therapies are urgently required.

Zeaxanthin is a carotenoid pigment, which belongs to the xanthophyll subclass, having a chemical formula C_40_H_56_O_2_. It is found at high levels in various foods (e.g., egg yolk, corn, and many vegetables and fruits), herbs, and traditional Chinese medications (*Hippophae rhamnoides, Lycium barbarum, Lycium chinense, Lilium hansonii, Cycas revolute,* and *Crocus sativus*) [5, 6]. It has been used in traditional Chinese medicine to treat various diseases and has been tested for its biomedical effects in several experimental disease models, including various cancers [7]. 

Humans are not capable of synthesizing zeaxanthin, and thus, the zeaxanthin content of the body is entirely dependent upon dietary intake [8, 9]. Zeaxanthin is present in various tissues and highly concentrated in the central retina (macula) of the eye [10–13]. Zeaxanthin is an antioxidant which also acts as a yellow filter protecting the macula from the blue light. Various observational and interventional studies have suggested that zeaxanthin might modify the risk of age-related macular degeneration (AMD) [14–19]. Therefore, supplementation of zeaxanthin with and without other antioxidants has been used clinically for the prevention and treatment of AMD with variable effects [20, 21].

Epidemiological studies have shown that higher intake and higher blood levels of zeaxanthin appear to be associated with a lower risk of occurrence of various cancers [22–44]. It has also been reported that zeaxanthin may inhibit cell growth or induce apoptosis of several tumor cell lines *in vitro* [45–48].

To our best knowledge, the effect of zeaxanthin on cultured uveal melanoma cells has not been reported previously. The purpose of the present study was to investigate the cytotoxic and apoptosis-inducing effects of zeaxanthin on human uveal melanoma cells *in vitro* compared to those in normal human uveal melanocytes, retinal pigment epithelial (RPE) cells, and fibroblasts. The effects of zeaxanthin on the Bcl-2 family of proteins, mitochondrial transmembrane potential (MTP), cystolic cytochrome c, and the activation of various caspases were also studied to elucidate the signaling pathway involved in zeaxanthin-induced apoptosis of uveal melanoma cells.

## 2. Materials and Method

### 2.1. Reagents

F-12 culture medium, Dulbecco's Modified Eagle's Medium (DMEM), fetal bovine serum (FBS), phosphate buffered saline (PBS), 0.05% trypsin-0.02% EDTA solution, and gentamicin were purchased from GIBCO (Grand Island, NY, USA). Isobutylmethylxanthine, cholera toxin, 3-[4,5-dimethylthiazol-2-yl]-2,5-diphenyltetrazolium bromide (MTT), and dimethyl sulfoxide (DMSO) were obtained from Sigma (St. Louis, MO, USA). Basic fibroblast growth factor was obtained from PeproTech (Rocky Hill, NJ, USA). Zeaxanthin was obtained from Dr. Dennis L. Gierhart (Chesterfield, MO, USA). Cytochrome c ELISA kit was purchased from Calbiochem (San Diego, CA, USA). Caspase-8 and -9 colorimetric activity assay kits were purchased from R&D Systems (Minneapolis, MN, USA). Caspase-3 colorimetric assay kits were purchased from Calbiochem. 

### 2.2. Cell Culture

The effects of zeaxanthin were tested in two human choroidal melanoma cell lines (SP6.5 and C918) and compared to effects on three different normal cell lines, human normal uveal melanocytes, fibroblasts, and retinal pigment epithelial (RPE) cells. SP6.5 melanoma cell line was isolated from a primary choroidal melanoma patient and was provided by Dr. Guy Pelletier (Research Center of Immunology, Quebec, Canada) [49]. Melanoma cell line C918 was derived from a choroidal melanoma patient with liver metastasis at the University of Iowa and was provided by Dr. Robert Folberg (University of Illinois, Chicago) and Dr. Xiaoliang Leon Xu (Memorial Sloan Kettering Cancer Center, New York) [50]. C918 is epithelioid in morphology and is a highly invasive, metastatic, and aggressive melanoma cell line [50]. The human uveal melanocytes (isolated from the choroid) and scleral fibroblasts were established in the Tissue Culture Center of the New York Eye and Ear Infirmary as previously reported [51]. The human RPE cell line, ARPE-19, was obtained from American Type Culture Collection (Manassas, VA, USA). All melanoma cells, RPE cells, and fibroblasts were cultured with DMEM, supplemented with 10% FBS and gentamicin (50 **μ**g/mL). Uveal melanocytes were cultured with FIC medium, supplemented with 10% FBS and gentamicin [51]. Cells were incubated at 37°C in a CO_2_ regulated incubator in a humidified 95% air/5% CO_2_ atmosphere. After cultures reached confluence, cells were detached with trypsin-EDTA solution and passaged. All tissues were obtained with premortem consent in accordance with the laws and regulations in place in the various jurisdictions.

### 2.3. MTT Assay for Cell Viability

Methods for MTT assay have been described previously [52]. Briefly, cells were seeded into 96-well plates at a density of 5 × 10^3^ cells per well. After 24 h, zeaxanthin was applied to the cultures. Zeaxanthin (6.82 mg) was dissolved in 200 *μ*L DMSO to make a stock solution of 60 mM. Tested cells were treated by different concentrations of zeaxanthin. The cells in the control group were cultured in medium containing the same levels of DMSO as in the zeaxanthin solution. A separate investigation about the effects of the highest DMSO levels (1 : 200) used in this experiment did not show significant differences in the cell viability between the cells with and without DMSO. After 48 h incubation with or without zeaxanthin, culture medium was aspirated and replaced with fresh culture medium. MTT solution (1 mg/mL, 50 *μ*L) was added to each well. After 4 h incubation at 37°C, the medium and MTT were aspirated and the formazan blue that formed in the cells was dissolved in 100 *μ*L of DMSO. Optical density of the 96-well plates was determined with a microplate reader (Multiskan MCC/340, Fisher Scientific, Pittsburgh, PA, USA) at 540 nm. The optical density of formazan formed in control (untreated) cells was taken as 100% viability. The concentration at which cell viability was inhibited by 50% (the 50% inhibitory concentration, IC_50_) was determined by linear interpolation. All tests were performed in three independent experiments.

To study the time-effect of zeaxanthin on uveal melanoma cells, melanoma cells (C918 cell line) were seeded into 96-well plates at a density of 5 × 10^3^ cells per well and divided into two groups, zeaxanthin-treated group and control group (without treatment of zeaxanthin). After 24 h, zeaxanthin was added to the cultures in the treated groups at final concentrations of 30 *μ*M. At 0, 6, 12, 24, and 48 h after the addition of zeaxanthin; MTT test was performed in both treated and control groups. The results of each treated group were compared to the controls cultured for the same time periods. All tests were performed in three independent experiments.

### 2.4. Flow Cytometric Detection of Apoptotic Cells

Cell apoptosis was measured using the Apoptotic/Necrotic Cells Detection Kit (Promokine, Heidelberg, Germany) and analyzed by flow cytometry. Uveal melanoma cells (SP6.5 and C918 cell lines) were seeded in 6-well plates at a density of 1.2 × 10^5^ cells per well. After treatment with zeaxanthin (30 *μ*M) for 6 h and 24 h, cells were washed in PBS, detached from the well using trypsin, centrifuged for 5 min to discard supernatant, and resuspended in binding buffer. FITC-annexin V and ethidium homodimer III were added to the suspension and incubated for 15 min at room temperature in darkness. Specimens, containing 1 × 10^5^ cells each, were then analyzed using a Becton Dickinson Flow Cytometer (Becton & Dickinson, Franklin Lakes, NJ, USA). The data were analyzed using Cell Quest V3.3 software and results were reported as percentages of the cell populations.

### 2.5. Western Blot Analysis for BCL-2 Family Proteins

Uveal melanoma cells (SP6.5 and C918 cell lines) were seeded in 10 cm culture dishes at a density of 6 × 10^6^ cells per dish. Zeaxanthin (30 *μ*M) was added to the medium 24 h later. After being cultured for 24 h, cells were washed in PBS and then scraped from the well, centrifuged for 5 min to discard the supernatant, and finally resuspended in binding buffer. Lysates were prepared by homogenizing cell pellets in lysis buffer. Protein concentrations were determined by the BCA protein assay kit (Novagen, Germany), using bovine serum albumin as the standard. Protein (20 *μ*g) was separated by a precast of 10% SDS-PAGE gel (Invitrogen, Carlsbad, CA, USA) and transferred onto polyvinylidene difluoride membranes. The membranes were blocked with 5% milk for 1 h at room temperature. The membranes were incubated with the primary antibodies of anti-Bak, Bax, Bad, Bcl-2, Bcl-xL (1 : 500 dilution; Santa Cruz Biotechnology, Dallas, TX, USA), and calnexin (1 : 1000 dilution; Abcam, Cambridge, MA, USA) at 4°C overnight. The membranes were then incubated with a secondary antibody (anti-mouse, 1 : 10000 dilution and anti-rabbit, 1 : 6000 dilution) (Sigma) for 1 h at room temperature and the protein was detected using a chemiluminescence method. Chemiluminescent signals were captured using the Las-4000 (Fujifilm, Tokyo, Japan). The signal of each protein was determined using ImageJ software (National Institutes of Health, Bethesda, MD, USA).

### 2.6. MTP Assessment (JC-1)

MTP was assessed using a Mitochondria Staining Kit which employed JC-1 (5,5′,6,6′-tetrachloro-1,1′,3,3′-tetraethylbenzimidazol-carbocyanine iodine), a sensitive fluorescent dye as the probe. Uveal melanoma cells (C918 cell line) were seeded into 8-well chamber slides at a density of 6 × 10^4^ cells/well. After 24 h, cells were treated with 30 *μ*M of zeaxanthin for another 24 h. JC-1 at a final concentration of 10 *μ*M was added and incubated for 15 min at 37°C in the dark. JC-1 and culture medium were aspirated and cells were washed three times. Cells were immediately observed by fluorescence microscopy using dual band-pass filters (at 490 nm excitation and 530 and 590 nm emission). The assay is based on the aggregation of the dye and its fluorescence in the mitochondria. In the undamaged mitochondria, the aggregated dye appears as red fluorescence located in the mitochondria, whereas in cells with damaged MTP, the dye remains as monomers in the cytoplasm with diffuse green fluorescence.

### 2.7. Cytochrome c Release Assay

Uveal melanoma cells (C918 cell line) were seeded into 6-well plates at a density of 4 × 10^5^ cells/well (4 × 10^4^ cells/cm^2^), and 24 h later, zeaxanthin was added at final concentrations of 0, 10, 30, and 100 **μ**M. Cells were harvested at 2 h after the addition of zeaxanthin. Cultures were washed with cold PBS and then scraped from the well. After cell counting and centrifugation at 1,500 rpm for 5 min at 4°C, the cell pellets were collected. Cells were lysed in a cold hypotonic cell lysis buffer (BioSource, Camarillo, CA, USA) and centrifuged at 1000 ×g for 10 min. The supernatant (cytosol and mitochondria) was collected and centrifuged at 10,000 ×g for 20 min. The pellets (mitochondria) and the supernatant (cytosol) were collected separately. The cytochrome c level in the cytosol was measured using a cytochrome c enzyme-linked immunosorbent assay (ELISA) kit and performed in accordance with the manufacturer's instructions. The level of cytochrome c was expressed as a percentage of the controls (cells cultured without zeaxanthin). This was performed in three independent experiments.

### 2.8. Caspase-3, Caspase-8, and Caspase-9 Colorimetric Assay

Melanoma cells (C918 cell line) were seeded into 6-well plates at a density of 4 × 10^5^ cells/well, and 24 h later zeaxanthin was added at final concentrations of 0, 10, 30, and 100 *μ*M. Two hours after the addition of zeaxanthin, cells were washed with cold PBS and collected. After cell counting and centrifugation at 1,500 rpm for 5 min at 4°C, the cell pellets were collected. Cells were lysed by using the cell extraction buffer (BioSource) with protease inhibitor cocktail (Sigma) and PMSF (BioSource), incubated on ice for 30 min, and vortexed for 30 sec. The lysates were centrifuged at 10,000 rpm for 10 min at 4°C. The supernatant was stored at −70°C until analysis. The caspase-8, caspase-9, and -3 levels in cell lysates were measured using individual specific colorimetric kits, according to the manufacturer's instructions. The optical density was read using a microplate reader (Multiskan EX) at 405 nm. The activities of caspase-8, caspase-9, and caspase-3 were expressed as a percentage of the controls (cells cultured without zeaxanthin). This was performed in three independent experiments. 

### 2.9. Statistics

Statistical significances of difference of means throughout this study were calculated by ANOVA one-way test in comparing data from more than two groups and Student's *t*-test in comparing data between two groups. The data was analyzed using SPSS statistical software (SPSS Inc., Chicago, IL, USA). A difference at *P* < 0.05 was considered statistically significant.

## 3. Results

### 3.1. Effects of Zeaxanthin on Cell Viability of Melanoma Cells and Various Normal Cells

**Figure 1:**
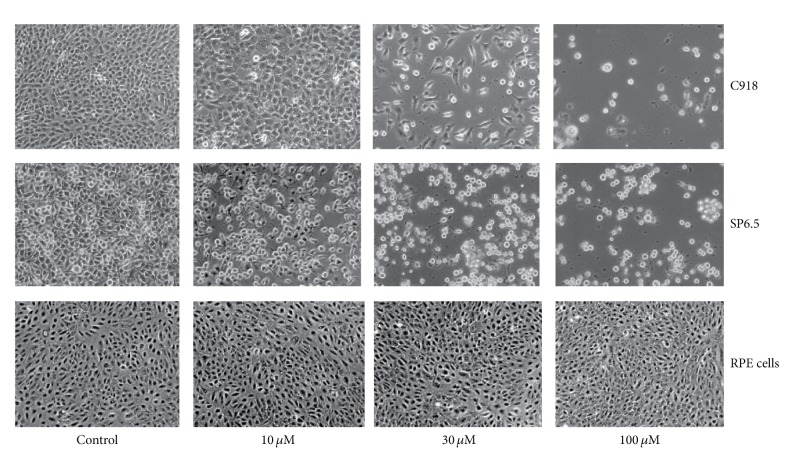
Phase-contrast photomicrograph of uveal melanoma cells and retinal pigment epithelial (RPE) cells treated with various concentrations of zeaxanthin. Human uveal melanoma cells (SP6.5 and C918) or RPE cells were seeded into 24-well plates at density of 8 × 10^4^/well (4 × 10^4^/cm^2^) and treated with zeaxanthin at 10, 30, and 100 **μ**M levels for 48 h. Zeaxanthin at all treated levels significantly decreased number of viable cells in melanoma cells, whereas, it did not affect the cell viability of RPE cells.

**Figure 2:**
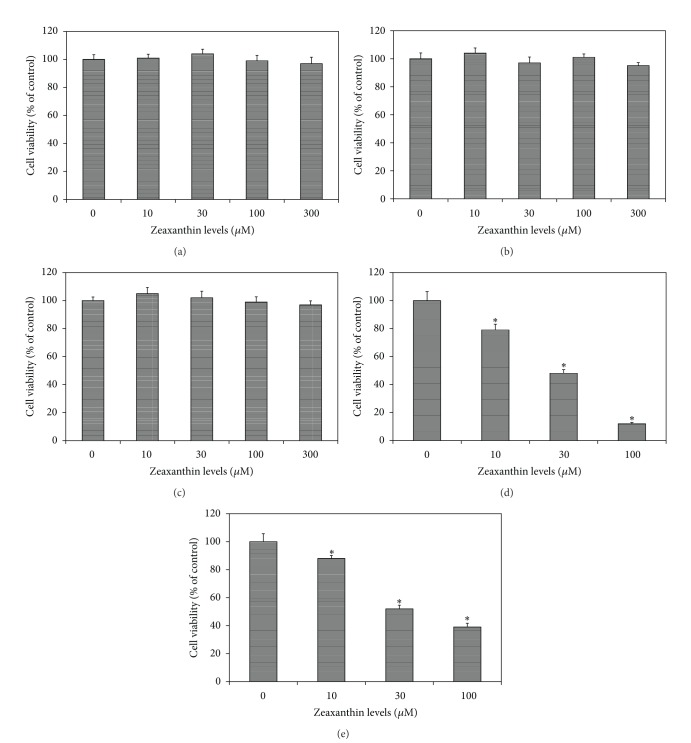
Dose effect of zeaxanthin on cell viability of uveal melanoma cells, uveal melanocytes, retinal pigment epithelial (RPE) cells, and fibroblasts. Human normal uveal melanocytes (a), RPE cells (b), and fibroblasts (c) or uveal melanoma cells C918 (d) and SP6.5 (e) were seeded into 96-well plates and treated with zeaxanthin at various doses for 48 h and cell viability was determined by MTT assay (see Section 2). Zeaxanthin at all tested concentrations selectively reduced the cell viability of uveal melanoma cells, without affecting cell viability of uveal melanocytes, RPE cells, and fibroblasts. Data are mean ± SD (*n* = 3). *P < 0.05, versus control (cells cultured without zeaxanthin).

Zeaxanthin at 10, 30, 100, and 300 **μ**M levels did not affect the cell viability of cultured human uveal melanocytes, RPE cells, or fibroblasts (Figures 1 and 2).

Zeaxanthin at 10, 30, and 100 *μ*M levels significantly decreased the cell viability of uveal melanoma cells in a dose-dependent manner (Figures 1 and 2). The difference in cell viability between cells treated with and without zeaxanthin at all tested levels (10, 30, and 100 *μ*M) was statistically significant (*P* < 0.05). The IC_50_ dose of zeaxanthin for cultured human uveal melanoma cells (SP6.5 and C918) at 48 h was 40.8 and 28.7 *μ*M, respectively. These results suggested that zeaxanthin at 10–100 *μ*M can selectively reduce the cell viability of melanoma cells without affecting normal uveal melanocytes, fibroblasts, or RPE cells. 

Time-effect study showed that zeaxanthin at 30 *μ*M decreased the cell viability of uveal melanoma cells (C918) in a time-dependent manner from 6 to 48 h (data not shown). The difference in cell viability at various time points, between cells treated with and without zeaxanthin, was statistically significant (*P* < 0.05).

### 3.2. Zeaxanthin-Induced Apoptosis of Melanoma Cells

**Figure 3:**
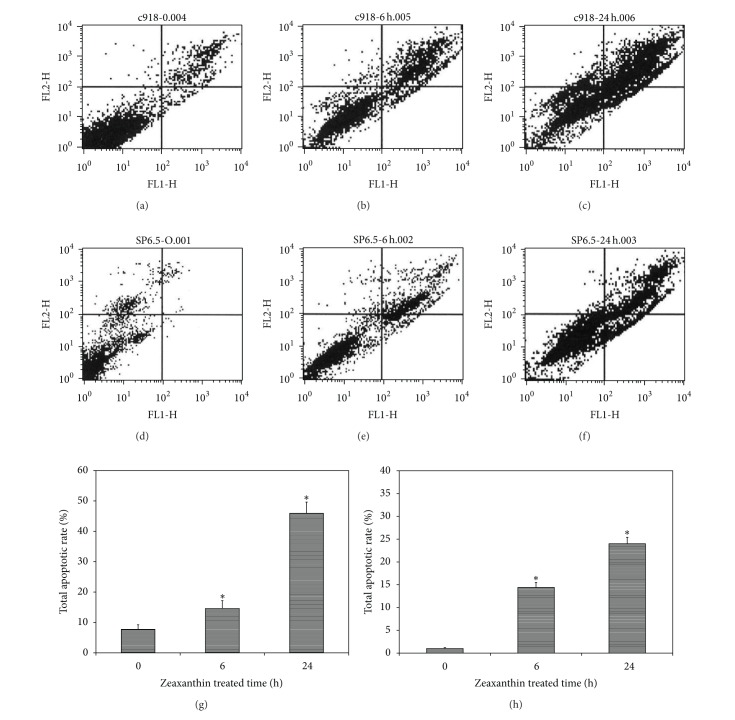
Effect of zeaxanthin on melanoma cell apoptosis as measured by annexin V-FITC/ethidium homodimer III (annexin/EtD III) flow cytometry. Melanoma cells (C918 and SP6.5) were treated by zeaxanthin at 30 **μ**M for 6 and 24 h, and the extent of apoptosis was determined by annexin/EtD III staining and analyzed by flow cytometry (see Section 2). (a)–(f) Annexin and EtD III negative cells are nonapoptotic cells (low left). Annexin positive and EtD III negative cells are in early stage of apoptosis (low right). Annexin and EtD III positive cells are in advanced stage of apoptosis (upper right). (a), (b), and (c) and (d), (e), and (f) are C918 and SP6.5 treated without zeaxanthin and treated with zeaxanthin at 6 and 24 h, respectively. Zeaxanthin significantly increased the rate of total apoptotic cells (the sum of early and advanced stage of apoptotic cells) in C918 (g) and SP6.5 (h). Data are mean ± SD (*n* = 3). *P < 0.05, versus control (cells cultured without zeaxanthin).

Uveal melanoma cells cultured with zeaxanthin (30 *μ*M for 6 and 24 h) and without zeaxanthin (controls) were double stained using annexin V and ethidium III and analyzed by flow cytometry. Nonapoptotic cells stained negatively with annexin. Apoptotic cells at early stage were annexin positive and ethidium-III negative, whereas apoptotic cells at advanced stage stained positively with both annexin and ethidium-III. Cells cultured with zeaxanthin for 6 and 24 h showed a significant increase in total apoptotic rate (*P* < 0.05, as compared with cells cultured without zeaxanthin) (Figure 3).

### 3.3. Western Blot Analysis for Effect of Zeaxanthin on BCL-2 Family Proteins

**Figure 4:**
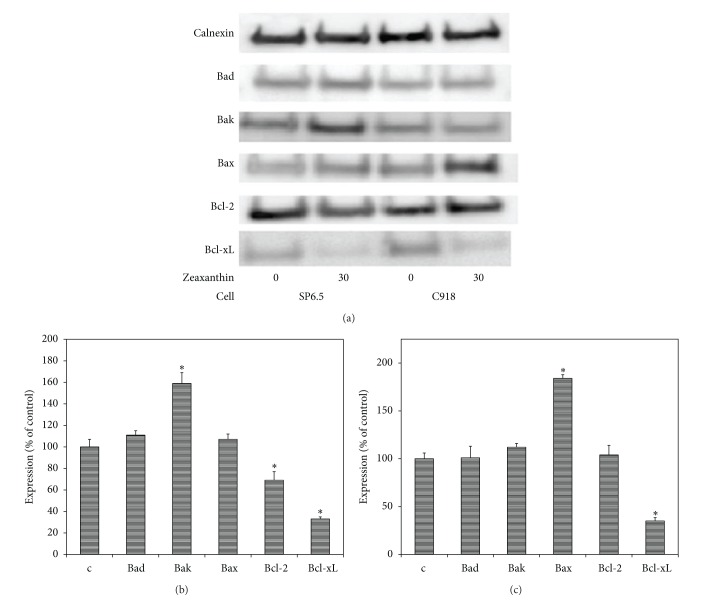
Western blot analysis of effects of zeaxanthin on expression of Bcl-2 family proteins in uveal melanoma cells. C918 and SP6.5 cell lines were treated with zeaxanthin (30 **μ**M) for 24 h. Cellular protein extracts were subjected to western blot analysis and probed with antibodies specific for proapoptosis member proteins (Bad, Bak, and Bax) and antiapoptosis member proteins (Bcl-2 and Bcl-xL). Calnexin was used as an internal loading control. Representative images are shown at (a). Quantitative results are presented at (b) (SP6.5 cell line) and (c) (C918 cell line). Data are mean ± SD (*n* = 3). *P < 0.05, versus control (cells cultured without zeaxanthin).

To assess the involvement of the Bcl-2 family of proteins in zeaxanthin-mediated apoptosis, Bax, Bak, Bad, Bcl-2, and Bcl-xL proteins, were examined in zeaxanthin-treated uveal melanoma cell lines. Western blot analysis revealed that zeaxanthin significantly decreased the expression of Bcl-xL and Bcl-2, antiapoptotic proteins, and increased the expression of Bak, a pro-apoptotic protein, in SP6.5 cells (*P* < 0.05 at zeaxanthin 30 *μ*M). Zeaxanthin significantly decreased the expression of Bcl-xL, an antiapoptotic protein, and increased the expression of Bax, a proapoptotic protein, in C918 cells (*P* < 0.05 at zeaxanthin 30 *μ*M) (Figure 4).

### 3.4. Effects of Zeaxanthin on MTP in Melanoma Cells

**Figure 5:**
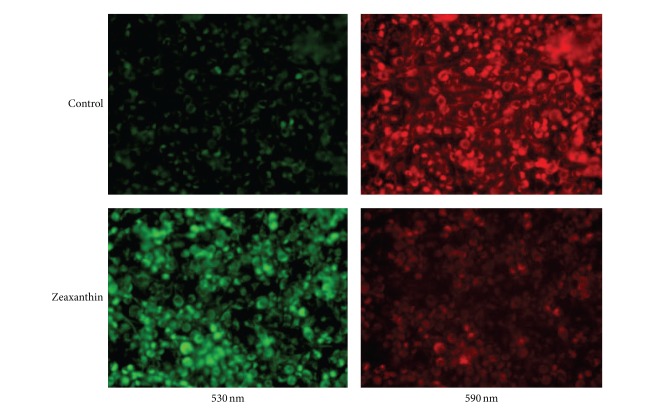
Fluorescence photomicrograph of effects of zeaxanthin on mitochondrial transmembrane potential (MTP) of uveal melanoma cells after JC-1 staining. Melanoma cells (C918) were treated by zeaxanthin at 0 (upper row) and 30 **μ**M (lower row) for 24 h, and the MTP was determined by JC-1 staining (see Section 2). Cells were observed by fluorescence microscopy using dual band-pass filters (at 490 nm excitation and 530 and 590 nm emission). In normal mitochondria, the aggregated dye appears as red fluorescence located in the mitochondria (upper), whereas in cells with damaged MTP, the dye remains as monomers in the cytoplasm with diffuse green fluorescence (lower). The MTP in melanoma cells was observed at 530 nm (left) and 590 mm (right).

In cells cultured with JC-1, red fluorescence is attributable to a potential-dependent aggregation of JC-1 in the mitochondria. Green fluorescence, reflecting the monomeric form of JC-1, appeared in the cytosol after mitochondrial membrane depolarization. As shown in Figure 5, uveal melanoma cells (C918) cultured without zeaxanthin exhibited mainly red fluorescence, indicating a normal MTP. Treatment with zeaxanthin (30 *μ*M) caused a diffuse green staining pattern, representative of damaged MTP (Figure 5).

### 3.5. Effects of Zeaxanthin on the Release of Cytochrome c into Cytosol

**Figure 6:**
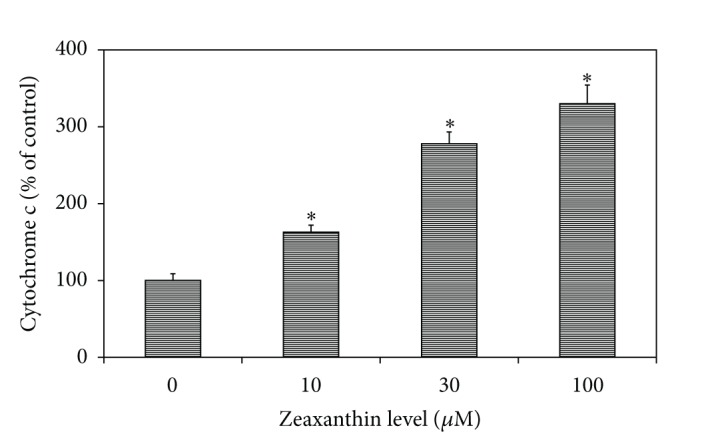
Effects of zeaxanthin-induced cytochrome c activation on human uveal melanoma cells. Uveal melanoma cells (C918) were treated with zeaxanthin at various doses for 2 h. Cells were collected and lysed, and the cytosol was extracted (see Section 2). The cytochrome c level in the cytosol was measured using a cytochrome c enzyme-linked immunosorbent assay kit. Cytosol cytochrome c level in zeaxanthin-treated cells at different concentrations was expressed as percentage of the controls. Zeaxanthin significantly increased the level of cytosol cytochrome c in a dose-dependent manner. Data are mean ± SD (*n* = 3). *P < 0.05, versus control (cells cultured without zeaxanthin).

Zeaxanthin increased cytosol cytochrome c levels in melanoma cells in a dose-dependent manner (Figure 6). Cytosol cytochrome c levels in cells treated with 10–100 *μ*M zeaxanthin were significantly increased (*P* < 0.05) as compared with the controls. In cells treated with 100 *μ*M zeaxanthin, cytosol cytochrome c level increased to 3.30-fold of the controls (cells cultured without zeaxanthin).

### 3.6. Effects of Zeaxanthin on Caspase-3, Caspase-8, and Caspase-9 Activities in Melanoma Cells

**Figure 7:**
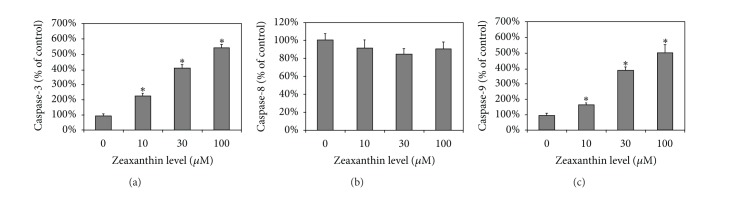
Effects of zeaxanthin on the activation of caspase-3, -8, and -9 in human uveal melanoma cell lines. Uveal melanoma cells (C918) were treated with various concentrations of zeaxanthin (10–100 **μ**M) for 2 h. Cells were collected and lysed (see Section 2). The caspase-3, -8, and -9 in the lysates were measured using relevant caspase colorimetric assay kits. Caspase-3, -8, and -9 levels in zeaxanthin-treated cells at different concentrations were expressed as percentage of the controls. Zeaxanthin significantly increased caspase-3 (a) and caspase-9 (c) activities in a dose-dependent manner, but not caspase-8 activities (b). Data are mean ± SD (*n* = 3). *P < 0.05, versus control (cells cultured without zeaxanthin).

Zeaxanthin significantly increased the caspase-3 and -9 activities in melanoma cells (C918) in a dose-dependent manner (Figures 7(a) and 7(c)). Both caspase-3 and -9 activities in uveal melanoma cells treated with 10–100 *μ*M zeaxanthin were very significantly increased (*P* < 0.05). A nearly 5.5-fold increase in caspase-3 and -9 activities was noted in uveal melanoma cells treated with 100 *μ*M zeaxanthin. Zeaxanthin at 10–100 *μ*M did not affect caspase-8 activities of uveal melanoma cells (Figure 7(b)).

## 4. Discussion

The relationship between the incidence of various malignant tumors and zeaxanthin in the diet or blood has been studied previously. Epidemiological studies showed that high intake and high blood levels of zeaxanthin (alone or combined with lutein) might be associated with a lower risk of occurrence of various malignant tumors, including non-Hodgkin lymphoma [22, 23], cervical [24–28], esophagus [29–31], stomach [31, 32], lung [33–35], breast [34, 36–38, 42], kidney [39], head and neck [40], colon [41, 43] and pancreas [31, 44], cancers. 

There were only a few reports on the effects of zeaxanthin on various cancer cells *in vitro* and these studies were tested only in few cancer cell lines. It has been reported that zeaxanthin might inhibit cell growth or cause apoptosis of lymphoma [45], breast cancer [45, 48], colon cancer [47], and neuroblastoma cells *in vitro* [46]. However, the mechanism of zeaxanthin-induced apoptosis has not been studied thoroughly. 

To our best knowledge, the relation between the intake and blood levels of zeaxanthin and the incidence of melanomas and the effect of zeaxanthin on cultured uveal melanoma cells has not been reported previously.

In the present study, zeaxanthin significantly decreased cell viability of two different human melanoma cell lines at concentration of 10–100 *μ*M in a dose-and time-dependent manner. The C918 cell line, which was isolated from a patient with choroidal melanoma and liver metastasis, is epithelioid in morphology and is a highly invasive, metastatic, and aggressive melanoma cell line [50]. C918 is more sensitive to cytotoxic effect of zeaxanthin as compared with the nonmetastatic melanoma cell line (SP6.5), indicating that zeaxanthin may be a promising candidate for prevention and treatment of metastatic uveal melanoma.

The effects of zeaxanthin on human uveal melanoma cells were compared with those in uveal melanocytes, fibroblasts, and RPE cells. Zeaxanthin at lower levels (10 *μ*M) significantly decreased the cell viability of melanoma cells, whereas the viability of these normal cells was not affected. Actually, the cell viability of normal cells was not affected at even the highest tested levels of zeaxanthin (300 *μ*M, which is 30-fold of the minimal toxic levels in melanoma cells), suggesting that zeaxanthin has specific anticancer activity in uveal melanoma cells.

Zeaxanthin is a hydrophobic molecule. Once ingested, zeaxanthin enters the liver through the portal vein and is taken up by the hepatocytes, incorporated into water-soluble lipoproteins, transported into the blood, and then transferred to various tissues [8, 9]. The distribution of zeaxanthin is eccentric among different tissues and organs. Ocular tissues have the highest levels of zeaxanthin, especially in the central retina, which can be several hundredfold the level in the serum [9]. The uveal tract also has a high level of zeaxanthin, accounting for up to 30% of the eye's total zeaxanthin content. The RPE and choroid may play a key role in the transport of zeaxanthin from circulating blood to the neural retina [12]. The zeaxanthin levels in mid-peripheral RPE and choroid are equal to 20–30% of the zeaxanthin levels in overlying retina [12]. Supplementation of zeaxanthin significantly increases serum and retina zeaxanthin levels [9–11]. In monkey zeaxanthin supplementation studies, the dietary supplementation of zeaxanthin at 2.2 mg/kg wt/day increased the serum zeaxanthin levels 10-fold [11, 53] and retina zeaxanthin levels from 4- to 9-fold in mid-peripheral and peripheral regions [10, 11]. Actually, supplementation safety dosages of zeaxanthin were far greater than the dosage (2.2 mg/kg wt/day) used in these experiments. One chronic study (52 weeks) in monkeys used 20 mg/kg/day and the dosage used in subacute studies in rats and mice was 50 mg/kg/day. (Gierhart, personal communication). Therefore, while the exact quantitative changes to zeaxanthin levels in uveal tract following supplementation are unknown, it is reasonable to assume that they are also significantly increased and may be comparable to those used in the present *in vitro* studies.

In the present study, the nature of cell death was studied using annexin V-ethidium-III double staining and flow cytometry analysis. An early event in apoptosis is a change in the phospholipid content of the cytoplasmic membrane outer leaflet. Phosphatidylserine (PS) is translocated from the inner to the outer surface of the cell. Annexin V is a phospholipid with a high affinity of PS. Annexin V labeled with fluorescein can bind to PS exposed on the outer leaflet and stain the cell membrane in bright green color [54]. Ethidium-III is a positively charged nucleic acid probe, which is impermeant to cells with intact plasma membrane. In the late stage of apoptosis, the cell membrane is damaged allowing ethidium-III to enter the cell and stain the nuclei producing red fluorescence. In the present study of the cells not treated with zeaxanthin, only very few of them stained with annexin and ethidium-III. Among cells treated with zeaxanthin, annexin-stained cells (indicating apoptotic changes) significantly increased compared to controls. The percentage of apoptotic cells also increased with time, suggesting that zeaxanthin can induce apoptosis of melanoma cells. This is consistent with previous reports which found an apoptosis-induction effect of zeaxanthin in breast cancer, colon cancer, lymphoma, and neuroblastoma cells [45–47]. 

There are two different apoptosis pathways, one extrinsic and one intrinsic. Extrinsic apoptosis occurs due to the activation of various cell death surface receptors following binding to the relevant ligands. Activated death receptors bind to secondary adaptor proteins which activate caspase-8 leading to a series of downstream events, including subsequent cleavage of caspase-3, and cell apoptosis. Intrinsic apoptosis pathway also called the mitochondrial apoptotic pathway is regulated by the Bcl-2 family of proteins, which includes the anti-apoptotic proteins (Bcl-2, Bcl-xL, etc.), the proapoptotic proteins (Bax and Bak) and the apoptosis initiator proteins (Bad, Bik, etc.). Accumulation of proapoptotic proteins on the mitochondrial outer membrane results in increased mitochondrial membrane permeability, causing the release of cytochrome c into the cytoplasm. Cytochrome c promotes activation of caspase-9, which in turn promotes activation of caspase-3, leading to apoptosis of the tumor cell [55–57].

Very little is known about the mechanism of zeaxanthin-induced apoptosis in malignant tumor cells [48]. In the present study, zeaxanthin reduced the expression of antiapoptotic proteins, Bcl-xL, in the two melanoma cell lines and increased the pro-apoptotic proteins (Bak) in SP6.5 cell line and Bax in C918 cell line. In additional, zeaxanthin decreased the Bcl-2 in SP6.5 cell lines. All of these results suggest that the zeaxanthin-induced apoptosis effect occurs via the intrinsic cell death pathway and is regulated by Bcl-2 family proteins. This finding is consistent with a previous report which found that zeaxanthin downregulated the expression of Bcl-2 mRNA in breast cancer cells [48].

The effects of zeaxanthin on the mitochondrial apoptosis signal pathway in malignant tumor cells have not been reported previously. In the present study, zeaxanthin significantly increased mitochondria outer membrane permeability as indicated by the mitochondrial staining test. This can lead to release of cytochrome c from mitochondria to cytosol, significantly increasing the cytosol cytochrome c levels, which in turn causes the activation of caspase-9 and -3 and results in the apoptosis. In this study, caspase-8, which plays an important role in the activation of the extrinsic apoptosis pathway, did not increase after exposing the melanoma cells to zeaxanthin. These results suggest that zeaxanthin induces apoptosis in uveal melanoma cells mainly via the intrinsic mitochondrial pathway.

Recently, it has been found that one of zeaxanthin's isomer, mesozeaxanthin [3,3′-dihydro-*β*,*β*-carotene], also has antimutagenic and anticarcinogenic potential. Mesozeaxanthin treatment increased survival of mice with 3-methylcholanthrene-induced sarcoma [58, 59].

In summary, zeaxanthin significantly decreased cell viability and induced apoptosis of human uveal melanoma cells without affecting the cell viability of normal cells, suggesting that zeaxanthin has a selective and potent pro-apoptotic effect on human uveal melanoma cells *in vitro*. This effect is mainly through the regulation of Bcl-2 family protein and intrinsic mitochondrial pathway. The potent and selective cytotoxic effects of zeaxanthin on a highly aggressive and metastatic human melanoma cell line (C918 cell line) suggest that zeaxanthin may be a promising agent worth exploring for the treatment of metastatic uveal melanoma.
